# IL-20 antagonist suppresses PD-L1 expression and prolongs survival in pancreatic cancer models

**DOI:** 10.1038/s41467-020-18244-8

**Published:** 2020-09-14

**Authors:** Shao-Wei Lu, Hong-Chin Pan, Yu-Hsiang Hsu, Kung-Chao Chang, Li-Wha Wu, Wei-Yu Chen, Ming-Shi Chang

**Affiliations:** 1 Institute of Basic Medical Sciences, College of Medicine, National Cheng Kung University, Tainan, Taiwan; 2 Department of Biochemistry and Molecular Biology, College of Medicine, National Cheng Kung University, Tainan, Taiwan; 3 Institute of Clinical Medicine, College of Medicine, National Cheng Kung University, Tainan, Taiwan; 4 Research Center of Clinical Medicine, National Cheng Kung University Hospital, College of Medicine, National Cheng Kung University, Tainan, Taiwan; 5 Department of Pathology, National Cheng Kung University Hospital, College of Medicine, National Cheng Kung University, Tainan, Taiwan; 6 Institute of Molecular Medicine, College of Medicine, National Cheng Kung University, Tainan, Taiwan; 7 Institute for Translational Research in Biomedicine, Kaohsiung Chang Gung Memorial Hospital, Kaohsiung, Taiwan

**Keywords:** Pancreatic cancer, Interleukins

## Abstract

Pancreatic ductal adenocarcinoma (PDAC) and cancer-associated cachexia (CAC) are multifactorial and characterized by dysregulated inflammatory networks. Whether the proinflammatory cytokine IL-20 is involved in the complex networks of PDAC and CAC remains unclear. Here, we report that elevated IL-20 levels in tumor tissue correlate with poor overall survival in 72 patients with PDAC. In vivo, we establish a transgenic mouse model (KPC) and an orthotopic PDAC model and examine the therapeutic efficacy of an anti-IL-20 monoclonal antibody (7E). Targeting IL-20 not only prolongs survival and attenuates PD-L1 expression in both murine models but also inhibits tumor growth and mitigates M2-like polarization in the orthotopic PDAC model. Combination treatment with 7E and an anti-PD-1 antibody shows better efficacy in inhibiting tumor growth than either treatment alone in the orthotopic PDAC model. Finally, 7E mitigates cachexic symptoms in CAC models. Together, we conclude IL-20 is a critical mediator in PDAC progression.

## Abstract (web summary)

The pro-inflammatory cytokine IL-20 promotes tumor growth in several cancer types. Here, the authors show that high levels of IL-20 are associated with poor survival in patients with pancreatic ductal adenocarcinoma (PDAC) and that IL-20 blockade reduces tumor growth and alleviates cachexia symptoms in mouse models of PDAC.

## Introduction

Pancreatic ductal adenocarcinoma (PDAC) often has a poor prognosis, even if it is diagnosed early. Oncogenic Kras mutation in mouse PDAC is the signature event and critical for tumor initiation^1^. Mutations in the proto-oncogene *KRAS* and tumor-suppressor gene *TP53* indicate a poor prognosis in PDAC^1^. The LSL-Kras^G12D^; Trp53^flox/flox^; Pdx-1-Cre (KPC) mouse model has been established as a clinically relevant PDAC model that develops many key features of human PDAC and a robust inflammatory response^2^. In the PDAC microenvironment, proinflammatory cytokines secreted by tumor cells and infiltrating immune cells, such as interleukin (IL)-1β, IL-6, and tumor necrosis factor (TNF)-α, have been shown to modulate PDAC progression and immune evasion^3^.

Tumor-associated macrophages (TAMs) are mainly differentiated from monocytes and recruited into tumors by cytokines^4^. In general, macrophages can be differentially polarized into a classical M1-like phenotype for inhibiting tumors or an alternative M2-like phenotype for promoting tumorigenesis and immunosuppression. Distinct from classical macrophages, TAMs are often functionally transformed by the tumor microenvironment and have an M2-like phenotype that promotes PDAC progression^5^. In addition, M2-like TAMs have been shown to contribute to immunosuppressive microenvironments in PDAC^6^. Inhibitors of immune checkpoint pathways have been linked to the promotion of tumor immune surveillance^7^. Programmed death-ligand 1 (PD-L1) is one of the ligands that binds to programmed death-1 (PD-1) on T cells and attenuates the immune response by downregulating the activity of antitumor T cells^8^. In the PDAC microenvironment, PD-L1 is highly expressed in cancer cells, facilitating immune escape and cancer progression^7^. Blockade of PD-L1 has been shown to inhibit tumor growth in a mouse model^9^. However, a previous study indicated that targeting PD-L1 for PDAC therapy was unsuccessful, as the response rate was <3.1% and there was no response to monotherapy^10^. In addition to its immunosuppressive microenvironment, PDAC is accompanied by tissue fibrosis, which facilitates cancer progression^11^. It has been shown that pancreatic tumors activate fibroblasts, leading to enhanced pancreatic tissue fibrosis^12^. In addition, the chronic activation of cancer-associated fibroblasts driven by the inflamed tissue microenvironment promotes pancreatic stromal remodeling that boosts tumor growth and progression^13^. Therefore, combined therapies that target tissue fibrosis pathways and enhance tumor immune surveillance may be important for treating PDAC.

During the progression of pancreatic cancer, cancer-associated cachexia (CAC), which involves progressive loss of body weight, wasting of the skeletal muscle, and atrophy of adipose tissues, often occurs in patients^14,15^. PDAC is often accompanied by CAC in later stages, which is the major cause of poor survival in these patients, and the 5-year survival rate is <5%^16,17^. CAC-induced fat loss is primarily due to increased lipolysis and the browning of white adipose tissue (WAT)^18^. The increased lipolysis is associated with the activation of hormone-sensitive lipase (HSL) and adipose triglyceride lipase (ATGL) in adipose tissue^19^. In patients, the anti-lipolytic effect of insulin on adipocytes is decreased, which stimulates lipolysis in CAC^20^. The browning of WAT is associated with increased expression of uncoupling protein 1 and increased lipid mobilization and energy expenditure during cachexia^21^. Inhibition of lipolysis and WAT browning has been shown to protect against CAC^21^. Proinflammatory cytokines, such as IL-1β, IL-6, TNF-α, and interferon (IFN)-γ, are crucial mediators of CAC, and targeting these cytokines has been suggested as a potential intervention for cachexia and prevention of metabolic imbalance^22^. However, the mechanisms underlying the crosstalk between cancer cells and tumor-associated immune cells during PDAC progression and CAC development remain elusive.

The proinflammatory cytokine IL-20 is a member of the IL-10 family, which includes IL-10, IL-19, IL-20, IL-22, IL-24, and IL-26. IL-20 is expressed predominantly by monocytes, dendritic cells, epithelial cells, and endothelial cells^23^. In addition, IL-20 receptor is constitutively expressed in epithelial cells and endothelial cells^24^. IL-20 has been shown to affect multiple cell types by activating a heterodimeric receptor complex of either IL-20R1/IL-20R2 or IL-22R1/IL-20R2 (ref. ^25^). Under physiological conditions, IL-20 is primarily involved in epidermal cell, keratinocyte, and monocyte differentiation. IL-20 induces signal transduction through the signal transducer and activator of transcription factor-3 pathway during keratinocyte proliferation and normal skin differentiation^24^. In immune regulation, IL-20 induces the production of chemokine ligand 2 by monocytes and regulates immune defense mechanisms^24,26^. Under pathological conditions, IL-20 expression is significantly upregulated in several inflammatory diseases, such as psoriasis, rheumatoid arthritis, atherosclerosis, renal failure, stroke, osteoporosis, and cancer^27–35^.

Our previous studies have demonstrated that IL-20 promotes tumor growth and increases metastasis in hepatocellular carcinoma, breast cancer, prostate cancer, and oral cancer^27–30^. We developed the anti-IL-20 monoclonal antibody (mAb) 7E, which was shown to neutralize the in vitro and in vivo activity of human and mouse IL-20, and showed that 7E treatment effectively alleviated inflammation and suppressed tumor growth in breast cancer and prostate cancer mouse models^28,29^. However, it is not clear whether IL-20 correlates with the progression of PDAC or whether targeting IL-20 could be an effective treatment for PDAC.

In this study, we investigate the role of IL-20 in PDAC and evaluate the efficacy of IL-20 blockade by 7E in an orthotopic tumor model and a KPC mouse model. We find that IL-20 blockade by 7E prolongs survival and alleviates pancreatic fibrosis in both mouse models, reduces the expression of the immunosuppressive molecule PD-L1 on tumor cells, inhibits tumor growth, and mitigates TAM infiltration. In addition, 7E treatment mitigates CAC manifestations, including reducing tumor-associated weight loss, maintaining the food intake, and reducing lipolysis in adipose tissue, in CAC models. Together, our results indicate that targeting IL-20 with 7E represents a therapeutic strategy for treating PDAC and CAC.

## Results

### IL-20 was correlated with PD-L1 level and survival in patients

**Fig. 1:**
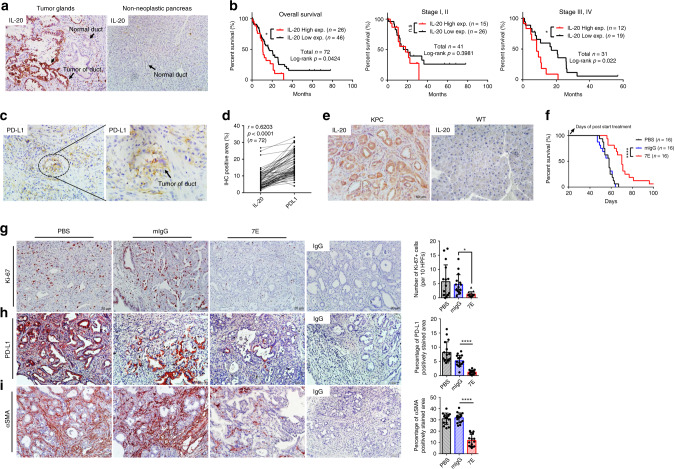
Poor clinical outcomes in pancreatic cancer were associated with high IL-20 expression in patients and targeting IL-20 prolonged survival and inhibited PD-L1 and αSMA expression in KPC mice. The expression of IL-20 or PD-L1 was analyzed in 72 clinical tissue samples by using immunohistochemical staining. **a** IL-20 staining was positive in tumor cells and neoplastic ductal epithelial cells in pancreatic tumors but negative in normal ducts (left panel). IL-20 staining was not visible in nonneoplastic ducts (right panel). Scale bar, 50 μm. **b** Kaplan–Meier analysis of the overall (left panel; *n* = 72 patients), early-stage (middle panel; *n* = 41 patients), and late-stage (right panel; *n* = 31 patients) survival of patients with PDAC with different levels of IL-20 expression was performed. *p* values, two-sided log-rank test. **c** PD-L1 staining was visible in the tumor tissue of a patient with PDAC, and the right image shows the enlarged area. Original magnification for images: ×200; scale bar, 50 μm; enlarged area: ×400, scale bar, 20 μm. **d** The expression of both IL-20 and PD-L1 was analyzed in PDAC tumors (*n* = 72 patients). Representative images of IL-20 and PD-L1 were analyzed by using the Tissue-Faxs software for IHC quantification and Pearson coefficients for the correlation between IL-20 and PD-L1. *p* < 0.0001. **e** IHC staining for IL-20 in pancreases from KPC and WT mice was performed. Original magnification: ×200; scale bar, 50 μm. **f** Kaplan–Meier survival analysis showed prolonged survival in 7E-treated KPC mice (*n* = 16). ****p < 0.0001 compared with mIgG-treated controls. **g** IHC analysis and quantification were performed for Ki-67^+^ proliferating cells in the pancreas of KPC mice treated with PBS, mIgG, or 7E (each group, *n* = 16). Original magnification: ×200; scale bar, 20 μm. One-way ANOVA, *p* = 0.0082; *p < 0.05 compared with mIgG-treated controls. IHC staining and quantification were performed for **h** PD-L1 (one-way ANOVA, *p* < 0.0001; *n* = 16) and **i** αSMA (one-way ANOVA, *p* < 0.0001; *n* = 15) expression in tumors from KPC mice. The rightmost panels show IgG-stained negative controls. Original magnification: ×200; scale bar, 50 μm. ****p < 0.0001 compared with mIgG-treated controls. Values represent the mean ± SD. Statistical significance was determined by log-rank test (**b**, **f**), two-tailed unpaired *t* test (**d**), or one-way ANOVA Sidak’s multiple comparisons test (**g**–**i**). The experiments in **a**, **c**, **e** were repeated three times independently with similar results, and the data of one representative IHC images are shown.

We performed immunohistochemical (IHC) staining to analyze the expression of IL-20 in tumor tissue samples from 72 patients with PDAC. IL-20 was strongly expressed in tumor cells and neoplastic ductal epithelial cells (Fig. 1a; left panel) but only lowly expressed by ductal epithelial cells in nontumorous pancreatic tissue (Fig. 1a; right panel). IL-20 expression was remarkably correlated with overall survival, and relatively high IL-20 expression predicted poor survival (Fig. 1b; left panel). There was a significantly negative correlation between high expression of IL-20 and survival in patients with advanced-stage PDAC (Fig. 1b; right panel). PD-L1 is highly expressed in PDAC and plays an important role in tumor progression. Thus we stained the 72 tumor tissue samples with an anti-PD-L1 antibody and compared the expression of IL-20 and PD-L1 in these patients. PD-L1 was expressed in the patients with PDAC (Fig. 1c), and IL-20 was significantly correlated with PD-L1 expression (*r* = 0.6203, *p* < 0.0001; Fig. 1d).

We determined the pathogenic role of IL-20 in vivo by using Pdx-1-Cre (KPC) mice, which spontaneously develop pancreatic tumors that mimic the progression of PDAC in humans^2,36^. IHC staining confirmed the upregulation of IL-20 expression in pancreatic tumor tissue samples from KPC mice compared with pancreatic tissue samples from wild-type (WT) mice (Fig. 1e). Neutralization of IL-20 with 7E (6 mg/kg, twice a week; treatment begun at the age of 25 days and continued until death) significantly prolonged the survival of KPC mice compared with control phosphate-buffered saline (PBS) or mouse immunoglobulin G (mIgG) treatment (Fig. 1f). In addition, 7E treatment effectively reduced the number of Ki-67^+^ proliferating cells in comparison to PBS and mIgG treatment (Fig. 1g).

PD-L1 is a ligand of PD-1 and expressed on tumor cells. Previous studies have shown that PD-L1 plays a role in regulating tumor growth, and this molecule is a new therapeutic target^37,38^. Pancreases from KPC mice treated with PBS or mIgG displayed prominent expression of PD-L1, and significant inhibition of PD-L1 expression was observed in 7E-treated mice, which had a longer life span than the mice in the two control groups (Fig. 1h).

Pancreatic fibrosis plays an important role in the pathogenesis of PDAC. We previously reported that IL-20 acts as a fibrogenic factor in liver fibrosis^39^. To investigate the effect of IL-20 on the pancreatic fibrosis occurring during PDAC, we evaluated the distribution of alpha-smooth muscle actin (αSMA), a hallmark of fibrosis. IHC analysis showed that αSMA was overexpressed in PBS- and mIgG-treated KPC mice, but αSMA expression was substantially reduced in 7E-treated KPC mice (Fig. 1i).

### IL-20 blockade inhibited orthotopic pancreatic tumor growth

**Fig. 2:**
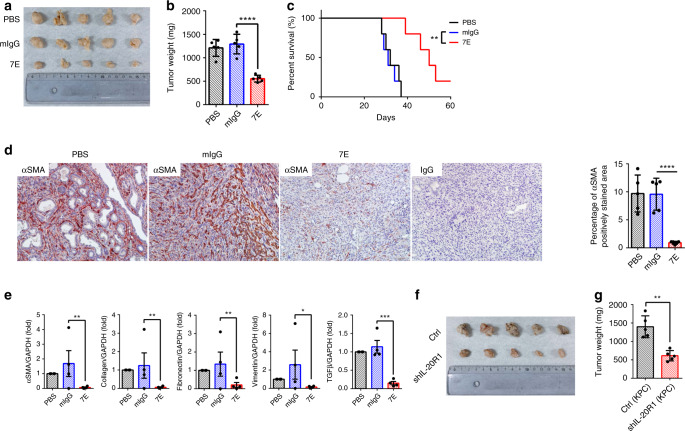
Tumor growth was inhibited in 7E-treated and IL-20R1-knockdown KPC cell-injected orthotopic models of PDAC. KPC cells were injected into the pancreas of WT mice, which were then treated (i.p.) with PBS, mIgG, or 7E (*n* = 5) twice per week for 28 days. Mice that were not injected with cancer cells served as healthy controls (*n* = 3). **a**, **b** The tumors were collected on day 28, measured, and weighed. One-way ANOVA, *p* < 0.0001. ****p < 0.0001 compared with mIgG-treated controls. **c** Mice were randomly divided into three groups (*n* = 5) and treated with PBS, mIgG, or 7E twice per week until the end of the study. Kaplan–Meier survival analysis showed prolonged survival for the 7E-treated orthotopic model mice. Log-rank *p* value = 0.0028; **p < 0.01 compared with mIgG-treated controls. **d** Representative IHC and quantification were performed for αSMA in PDAC tumors from KPC mice (each group, *n* = 5). Original magnification: ×200; scale bar, 50 μm. The rightmost panel shows the IgG-stained negative control. One-way ANOVA, *p* < 0.0001. **e** Transcript levels of αSMA (one-way ANOVA, *p* = 0.0099), Collagen (one-way ANOVA, *p* = 0.011), Fibronectin (one-way ANOVA, *p* = 0.0144), Vimentin (one-way ANOVA, *p* = 0.0282), and TGF-β (one-way ANOVA, *p* < 0.0001) were determined by RT-qPCR analysis of pancreatic tumors (each group, *n* = 4). *p < 0.05, **p < 0.01, and ***p < 0.001 compared with mIgG-treated controls. **f**, **g** KPC or IL-20R1-knockdown KPC cells were injected into the pancreas of WT mice (*n* = 5). Tumor images are shown (**f**), and tumors weights (**g**) were measured. **p = 0.0079 compared with the control group. Data are shown as the mean ± SD in **b**–**d**, **g**. Data are shown as the mean ± SEM in **e**. Statistical significance was determined by one-way ANOVA Sidak’s multiple comparisons test (**b**, **d**, **e**), log-rank test (**c**), or Mann–Whitney *U* test (**g**).

We next investigated whether IL-20 blockade by 7E inhibits orthotopic pancreatic tumor growth. To test this, pancreatic tumor cells isolated from KPC mice were transfected with a luciferase reporter gene (KPC/Luc cells). 7E treatment significantly inhibited tumor growth in vivo, as measured on day 28 after inoculation of KPC/Luc cells (Fig. 2a). The tumor sizes and weights in the 7E-treated group were significantly smaller than those in the mIgG- and PBS-treated groups (Fig. 2a, b). In line with the findings in transgenic KPC mice, 7E treatment prolonged survival and decreased αSMA expression compared with PBS or mIgG treatment in the orthotopic model (Fig. 2c, d). Furthermore, 7E treatment inhibited the expression of fibrogenesis-related markers, including αSMA, Collagen, Fibronectin, Vimentin, and transforming growth factor (TGF)-β (Fig. 2e).

For both KPC mice and orthotopic PDAC model mice, we performed enzyme-linked immunosorbent assay (ELISA) analyses to measure the circulating levels of IL-20 and found that the serum levels of IL-20 were significantly higher in the KPC mice and orthotopic PDAC model mice than the corresponding healthy control groups. The serum levels of IL-20 were reduced in 7E-treated mice for both models. These data indicated that IL-20 was not only highly expressed in tumor tissues but also expressed systemically (Supplementary Fig. 1a, b).

### Inhibiting IL-20 attenuated cancer cell proliferation

To clarify the cellular sources of IL-20 receptors in the pancreatic tumor microenvironment, we performed IHC staining to analyze the expression of IL-20 receptors (IL-20R1, IL-20R2, and IL-22R1) in tumor and nontumor stromal tissue samples from patients and mice with pancreatic cancer. Positive staining for IL-20 receptors was observed on tumor cells but not on the stromal cells in nontumor pancreatic tissue (Supplementary Fig. 2a). Immunostaining showed that both human and murine pancreatic cancer cell lines (PANC-1, BxPC-3, and PANC-02) expressed IL-20 and its receptors (Supplementary Fig. 2b). A methyl thiazol tetrazolium (MTT) assay showed that an anti-IL-20 mAb (7E) suppressed IL-20-mediated cell proliferation in these three pancreatic cancer cell lines (Supplementary Fig. 2c–e), suggesting that IL-20 is pivotal in pancreatic cancer cell proliferation.

### IL-20R1 knockdown in KPC cells inhibited tumor growth

Inhibition of IL-20 by 7E reduced tumor growth in vivo (Figs. 1, 2), which may be attributed to reductions in the autocrine effects of IL-20 within the tumor microenvironment. To further confirm the critical role of IL-20-mediated signaling (IL-20R1) in tumor growth, we generated IL-20R1-knockdown KPC cells (Supplementary Fig. 3) and orthotopically injected these cells into the pancreas of C57BL/6 mice. Tumor volume and weight were significantly decreased in the IL-20R1-knockdown KPC tumor group compared with the parental KPC tumor group (Fig. 2f, g), suggesting that IL-20R1 directly contributes to the tumorigenic activity of KPC cells.

### IL-20 blockade inhibited M2 macrophage in vivo and in vitro

**Fig. 3:**
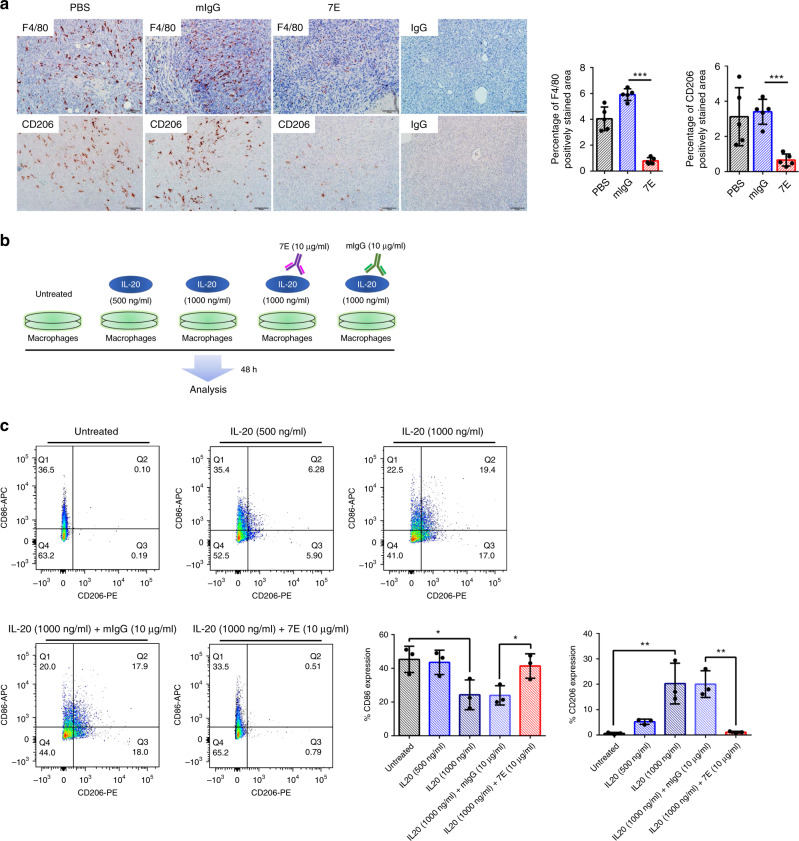
7E decreased macrophage infiltration in an orthotopic model of pancreatic cancer. KPC cells were injected into the pancreas of mice. PBS, mIgG, or 7E treatment was performed (*n* = 5), and tumors were harvested at day 28. **a** IHC staining for F4/80 (one-way ANOVA, *p* = 0.0131) and CD206 (one-way ANOVA, *p* < 0.0001) counterstaining in a tumor tissue sample. Immunohistochemical quantification with the HistoQuest software (right) demonstrated a reduction in infiltrating macrophage numbers in the tumor (*n* = 5). Original magnification: ×200; scale bar, 50 μm. The rightmost panel shows the IgG-stained negative control. ***p < 0.001 compared with the mIgG-treated controls. **b** Flowchart of treatment. BMDMs were treated with IL-20 for 48 h, and 7E was used to neutralize the activity of IL-20. **c** The number of CD206 (one-way ANOVA, *p* = 0.0003) and CD86 (one-way ANOVA, *p* = 0.0089) in macrophages was determined by flow cytometry. The results in the bar graph (right) represent the average value of three independent experiments. *p < 0.05, **p < 0.01. Data are presented as mean ± SD. Statistical significance was determined by one-way ANOVA Sidak’s multiple comparisons test (**a**, **c**).

TAMs play a major role in tumor progression. To investigate whether blockade of IL-20 affects macrophages in pancreatic tumors, we stained tumor tissue samples for a macrophage marker (F4/80) and an M2-type macrophage marker (CD206). The frequencies of F4/80^+^ macrophages and CD206^+^ macrophages were decreased in 7E-treated mice (Fig. 3a). These results suggested that inhibiting IL-20 might reduce the numbers of M2-type macrophages in pancreatic tumors in the orthotopic model. To evaluate whether IL-20 induces M2 macrophage differentiation in vitro, we incubated bone marrow-derived macrophages (BMDMs) with mIL-20 for 48 h and analyzed macrophage polarization (Fig. 3b). Fluorescence-activated cell sorting analysis showed that IL-20 dose-dependently upregulated the CD206^+^ macrophage frequency and that 7E inhibited IL-20-induced CD206^+^ macrophage polarization (Fig. 3c and Supplementary Fig. 4). Conversely, blockade of IL-20 increased CD86^+^ (M1-type macrophage marker) macrophage differentiation (Fig. 3c).

### IL-20 upregulated PD-L1 in tumors of the orthotopic model

**Fig. 4:**
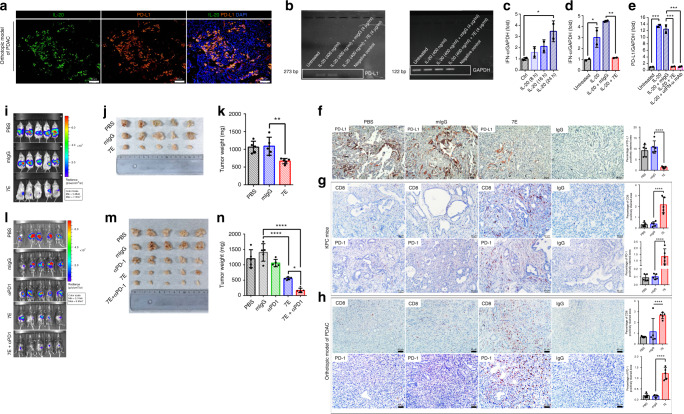
IL-20 induced PD-L1 expression, and 7E neutralized IL-20-induced PD-L1 expression. **a** Pancreatic tissue from an orthotopic PDAC model mouse was analyzed by immunofluorescence labeling of IL-20 (green) and PD-L1 (red). Nuclei (blue) were visualized by DAPI staining. Scale bar, 50 μm. **b** KPC cells were treated with IL-20 or IL-20 plus 7E, and the expression of PD-L1 in the KPC cells was analyzed using RT-PCR with specific primers. **c** KPC cells were treated with mIL-20 for the indicated time, and the fold increase in the expression of IFN-α was analyzed using RT-qPCR with specific primers (*n* = 1 strain of cell culturing; one-way ANOVA, *p* = 0.0488). **d** IFN-α mRNA expression (*n* = 1 strain of cell culturing; one-way ANOVA, *p* < 0.0001) and **e** PD-L1 mRNA expression in KPC cells were analyzed after IL-20 treatment using RT-qPCR (*n* = 1 strain of cell culturing; one-way ANOVA, *p* = 0.0042). *p < 0.05, **p < 0.01, and ***p < 0.001. KPC mice and orthotopic PDAC model mice were treated with 7E, and pancreatic tissue samples were analyzed for **f** PD-L1 (one-way ANOVA, *p* < 0.0001), **g**, **h** CD8, and PD-1 staining quantification. The rightmost panel shows the IgG-stained negative control. Scale bar, 50 μm. **g** CD8 and PD-1: one-way ANOVA, *p* < 0.0001. **h** CD8 and PD-1: one-way ANOVA, *p* < 0.0001. ****p < 0.001 compared with the mIgG-treated controls. KPC/Luc cells were injected into the pancreas of WT mice. PBS, mIgG, an anti-PD-1 antibody, 7E, or 7E + the anti-PD-1 antibody (*n* = 5) were injected (i.p.) into the mice twice per week for 28 days. **i** Analysis of tumor growth by using the in vivo luminescence activity of KPC/Luc cells on day 21. **j**, **k** Tumors were collected and weighed (*n* = 5; one-way ANOVA, *p* = 0.005). **l** Luciferase activity was measured on day 21 posttreatment and used to quantify tumor growth. **m**, **n** Tumors were harvested and analyzed (*n* = 5; one-way ANOVA, *p* < 0.0001). *p < 0.05, **p < 0.01, and ****p < 0.0001. Values represent the mean ± SD. Statistical significance was determined by one-way ANOVA Sidak’s multiple comparisons test (**c**–**h**, **k**, **n**). The experiments in **c**–**e** were repeated three times independently with similar results, and the data of one representative experiment are shown.

To examine the relationship between IL-20 and PD-L1, we used co-immunofluorescence staining to analyze the expression of IL-20 and PD-L1. Pancreatic tumors from the orthotopic model showed colocalization of IL-20 and PD-L1 in vivo, which indicated that IL-20 might be involved in PD-L1 regulation (Fig. 4a). We further investigated whether PD-L1 is regulated by IL-20 in vitro. KPC cells were treated with IL-20, and reverse transcription polymerase chain reaction (RT-PCR) showed that PD-L1 expression was upregulated in the IL-20-treated KPC cells and that 7E blocked the activity of IL-20 (Fig. 4b). A recent publication reported that IFN-α is correlated with PD-L1 induction^40^. To elucidate whether IL-20 upregulates PD-L1 expression through activation of IFN-α, RT-qPCR was performed and revealed that IL-20 upregulated the expression of IFN-α and that 7E blocked IL-20-induced IFN-α expression (Fig. 4c, d). Inhibiting the activity of IFN-α with an anti-IFN-α antibody completely inhibited PD-L1 expression in IL-20-treated KPC cells, indicating that IL-20 regulates PD-L1 expression via IFN-α activation (Fig. 4e). Based on our in vitro data, we speculated that blockade of IL-20 would suppress PD-L1 expression in vivo. PD-L1 expression was weaker in 7E-treated mice than in PBS- and mIgG-treated mice (Fig. 4f). Overall, these results are consistent with the in vitro data and confirmed that 7E reduces PD-L1 expression in vivo and in vitro.

### IL-20 blockade promoted CD8^+^ T cell infiltration

Previous studies demonstrate elevated expression of the checkpoint receptor PD-1 in activated T cells in PDAC^41,42^. To address whether the reduction in tumor growth observed in 7E-treated mice is also mediated by T cells, we performed IHC staining with an anti-CD8 antibody, the cytotoxic T cell marker, on tumor tissue samples from orthotopic PDAC model and KPC mice. In these two models, increased CD8 and PD-1 expression was observed in 7E-treated mice but not in PBS- or mIgG-treated mice, indicating that 7E treatment promoted CD8^+^ T cell infiltration into tumor tissues (Fig. 4g, h). To further clarify whether 7E can restrain tumor growth in T cell-deficient mice, we generated an orthotopic model of PDAC in NOD.Cg-Prkdc^scid^Il2rg^tm1Wjl^/YckNarl mice (advanced severe immunodeficiency (ASID)) in which T cells, B cells, and natural killer (NK) cells are absent. We found that the tumor size and tumor weight of 7E-treated mice were still smaller than those of PBS- and mIgG-treated mice (Fig. 4i–k), indicating that IL-20 blockade can directly inhibit tumor growth in a T cell-, B cell-, and NK cell-independent manner.

### 7E combined with anti-PD-1 antibody showed better efficacy

Based on the findings that inhibiting IL-20 enhanced the infiltration of CD8^+^ and PD-1^+^ cells in orthotopic PDAC model and KPC mice, we postulated that combined treatment with IL-20 blockade by 7E and immune checkpoint inhibition with an anti-PD-1 antibody, which enhances CD8^+^ T cell activity, could produce synergistic efficacy for pancreatic cancer treatment. In the orthotopic PDAC model, we indeed observed that tumor weight was decreased more in mice treated with the combination of 7E plus the anti-PD-1 antibody than in mice treated with either 7E or the anti-PD-1 antibody alone (Fig. 4l–n).

### IL-20 blockade alleviated cachexia in the KPC tumor model

**Fig. 5:**
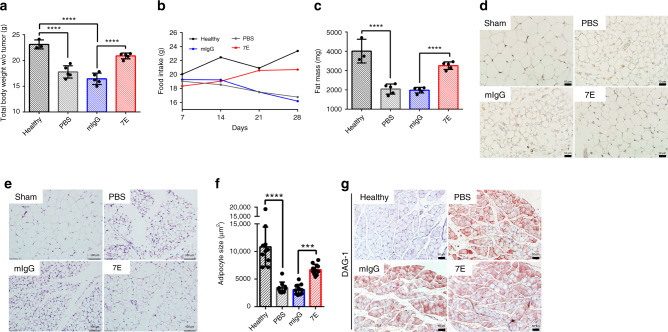
7E reversed fat mass loss in an orthotopic model of pancreatic cancer. KPC/Luc cells were injected into the pancreas of mice. PBS, mIgG, or 7E (*n* = 5) was injected (i.p.) into the mice twice per week for 28 days. Mice not injected with cancer cells served as healthy controls (*n* = 3). **a** The tumor-free body weight of the mice was measured on day 28. One-way ANOVA, *p* < 0.0001; ****p < 0.0001. **b** The food intake of the mice was monitored every week. Data are shown as the mean. **c** Epididymal fat was excised and weighed. One-way ANOVA, *p* < 0.0001; ****p < 0.0001. **d** IHC staining for F4/80+ macrophages in the epididymal fat of orthotopic model mice was performed. Original magnification: ×400; scale bar, 10 μm. **e** Representative images of epididymal fat sections stained with H&E for histological analysis are shown. Original magnification: ×200; scale bar, 100 μm. **f** Adipocyte size was quantified from randomly selected 10 cells (*n* = 10 cells examined). One-way ANOVA, *p* < 0.0001; ***p < 0.001; ****p < 0.0001. **g** IHC staining for DAG-1 in the soleus muscle of orthotopic model mice was performed. Original magnification: ×200; scale bar, 50 μm. Values represent the mean ± SD. Statistical significance was determined by one-way ANOVA Sidak’s multiple comparisons test (**a**, **c**, **f**).

7E treatment or knockdown of IL-20R1 signaling profoundly inhibited orthotopic KPC tumor growth (Fig. 2). We therefore hypothesized that blocking IL-20 ameliorates CAC. Tumor-bearing mice reduced their food intake and lost body weight compared with tumor-free healthy mice (Fig. 5a, b). Interestingly, the body weight and food intake of tumor-bearing mice treated with 7E continued to increase over time, as seen in the healthy mice (Fig. 5a, b). In addition, 7E treatment prevented the cancer-induced loss of epididymal fat mass (Fig. 5c), reduced infiltration of macrophages into adipose tissue (Fig. 5d), and preserved adipose tissue size (Fig. 5e, f).

Most cancer cachexia is accompanied by muscle wasting, in which the expression of the muscle wasting marker β-dystroglycan (DAG-1) is enhanced in muscle tissues^43^. DAG-1 was prominently expressed in the soleus muscle of mice in the PBS- and mIgG-treated groups in the orthotopic model (Fig. 5g). However, 7E treatment did not decrease DAG-1 expression, and there was no significant difference among these three groups. These data suggest that IL-20 may not be directly involved in cancer-induced muscle wasting.

### Anticachexia effect of 7E in Lewis lung carcinoma (LLC) model

**Fig. 6:**
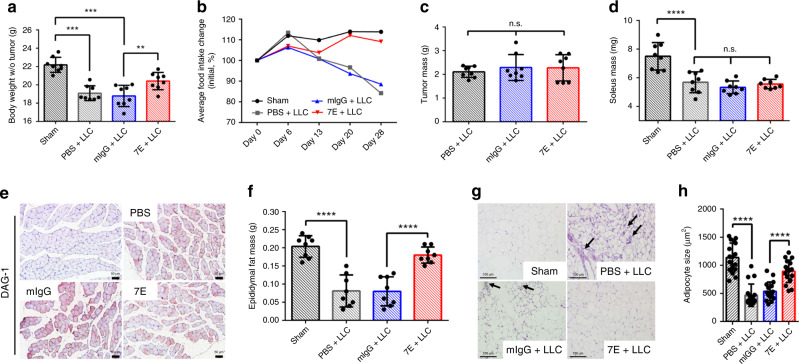
7E treatment alleviated LLC tumor-induced cachexia symptoms in mice. LLC tumor-bearing mice were injected (i.p.) with PBS, mIgG, or 7E (*n* = 8) twice per week throughout the study. Sham controls (*n* = 8) were not injected with cancer cells. **a** The tumor-free body weight of the mice was measured on day 28. One-way ANOVA, *p* < 0.0001; **p < 0.01, ***p < 0.001. **b** The average food intake of the mice was monitored every week. **c** Tumors were collected and weighed on posttreatment day 28. One-way ANOVA, *p* = 0.7059. **d** The soleus muscles were excised and weighed. One-way ANOVA, *p* < 0.0001; ****p < 0.0001. **e** IHC staining for DAG-1 in the soleus muscle of LLC model mice was performed. Original magnification: ×200; scale bar, 50 μm. **f** Epididymal fat was excised and weighed. One-way ANOVA, *p* < 0.0001; ****p < 0.0001. **g** Representative images of epididymal fat sections stained with H&E for histological analysis are shown. Original magnification: ×200; scale bar, 100 μm. Arrows indicate infiltrating immune cells. **h** Adipocyte size was quantified from randomly selected 17 cells (*n* = 17 cells examined). One-way ANOVA, *p* < 0.0001; ****p < 0.0001. Values represent the mean ± SD. Statistical significance was determined by one-way ANOVA Sidak’s multiple comparisons test (**a**, **c**, **d**, **f**, **h**).

We next explored whether IL-20 is a critical and common mediator in another mouse model of CAC, which is induced by inoculation of LLC cells^19^. Mice inoculated with LLC tumor cells lost significantly more body weight than sham control mice, while 7E treatment prevented cancer-induced body weight loss (Fig. 6a) and preserved the average food intake (Fig. 6b) compared with PBS or mIgG treatment. Intriguingly, 7E treatment did not reduce the LLC tumor mass (Fig. 6c) or cancer-induced muscle wasting in this model (Fig. 6d, e). Consistent with the findings in the orthotopic KPC tumor model, the epididymal fat mass was significantly preserved in the 7E-treated mice compared with the mIgG-treated mice (Fig. 6f). For LLC tumor-bearing mice, the size of adipocytes in epididymal fat was maintained in the 7E-treated group but not in the PBS- or mIgG-treated groups (Fig. 6g, h).

### 7E prevented fat loss and reduced serum triglyceride (TAG) levels

**Fig. 7:**
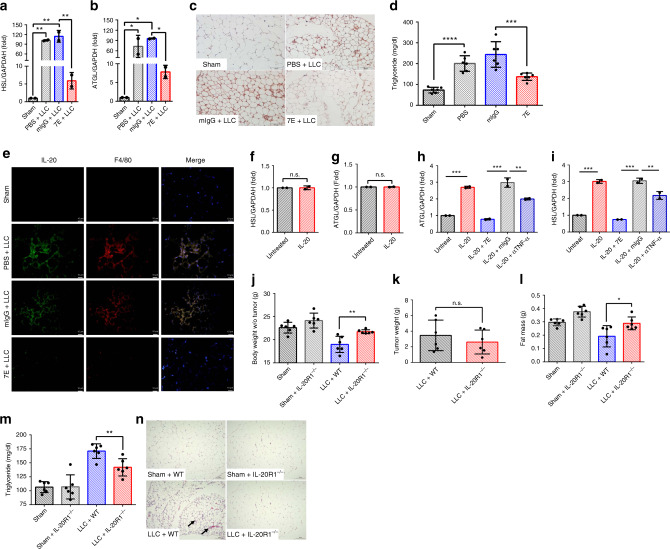
7E alleviated LLC tumor-induced cachectic lipolysis in mice, and IL-20R1 deficiency mitigated LLC tumor-induced cachexia symptoms. LLC tumor-bearing mice were injected (i.p.) with PBS, mIgG, or 7E (*n* = 6) twice per week throughout the study. Sham controls (*n* = 6) were not injected with cancer cells. The mRNA transcript levels of **a** HSL (one-way ANOVA, *p* = 0.0004) and **b** ATGL (one-way ANOVA, *p* = 0.0098) in the epididymal fat of LLC tumor-induced cachectic mice were analyzed using RT-qPCR. *p < 0.05, **p < 0.01. **c** IHC staining for HSL^+^ adipocytes in the epididymal fat of cachectic mice was performed. Scale bar, 50 μm. **d** The serum levels of triglycerides in LLC model mice were measured on day 28. One-way ANOVA, *p* = 0.0098; ***p < 0.001, and ****p < 0.0001. **e** Immunofluorescence staining showed the colocalization of IL-20 (green) and F4/80^+^ macrophages (red) in epididymal fat tissue samples. Nuclei were stained with DAPI (blue). Original magnification: ×400; scale bar, 10 μm. **f**, **g** The mRNA transcript levels of HSL and ATGL in adipocytes were analyzed using RT-qPCR. **h**, **i** RT-qPCR showed that conditioned medium from IL-20-treated LLC cells upregulated the expression of HSL and ATGL and that conditioned medium from 7E- or anti-TNF-α antibody-treated LLC cells inhibited IL-20-induced HSL and ATGL expression. One-way ANOVA, *p* < 0.0001; **p < 0.01, ***p < 0.001. LLC tumor cells were injected (s.c.) into the right flank of WT (*n* = 6) and IL-20R1^−/−^ mice (*n* = 6). On day 26 posttreatment, the **j** tumor-free body weight (Mann–Whitney *U* test, *p* = 0.022), **k** tumor weight, and **l** epididymal fat mass (Mann–Whitney *U* test, *p* = 0.0152) of the mice were determined. **m** Serum levels of triglycerides were measured by ELISA. Mann–Whitney *U* test, *p* = 0.0087. **p < 0.01. **n** Epididymal fat sections from the mice were stained with H&E for histological analysis. Arrows indicate immune cell infiltration. Scale bar, 100 μm. Data are shown as the mean ± SD. Statistical significance was determined by one-way ANOVA Sidak’s multiple comparisons test (**a**, **b**, **d**, **h**, **i**), paired *t* test (**f**, **g**), or Mann–Whitney *U* test (**j**, **l**, **m**). The experiments in **a**, **b**, **f**–**i** were repeated three times independently with similar results, and the data of one representative experiment are shown.

Browning of WAT, in which brown adipocytes are induced within WAT depots, and increased lipolysis are two major causes of CAC-induced fat loss^21,44^. The expression of the adipose browning-related genes *Ucp1*, *Pgc1a*, *Dio2*, and *Prdm16* was upregulated in the epididymal fat of LLC tumor-bearing mice^45^, whereas no difference in the expression of these genes was detected between the 7E- and mIgG-treated groups (Supplementary Fig. 5a–d). Thus we next analyzed whether 7E alleviates fat loss in the LLC-induced CAC model via inhibition of lipolysis. Increased lipolysis is associated with activation of the lipolytic enzymes HSL and ATGL in adipose tissue^46,47^. 7E treatment significantly reduced CAC-induced HSL and ATGL gene expression in epididymal fat following LLC cell inoculation (Fig. 7a, b). IHC staining confirmed that the increased number of HSL-positive cells in the epididymal fat of tumor-bearing mice was reduced by 7E treatment compared with PBS or mIgG treatment (Fig. 7c). Elevated levels of TAG and cholesterol are often detected in patients suffering from CAC^48^. Murine models of CAC also show increased serum levels of fatty acids and glycerol, suggesting increased TAG catabolism and delipidation of fat reserves^19^. In our LLC tumor-bearing mice, the level of TAG was significantly higher in the PBS- and mIgG-treated groups than in the sham control group, but the level of TAG was reduced in the 7E-treated group (Fig. 7d). The results suggest that IL-20 is involved in abnormal lipid metabolism during CAC but this activity can be neutralized with the anti-IL-20 antibody.

### 7E reduced F4/80^+^ macrophage infiltration to adipose tissue

Macrophages play an important role in the pathogenesis of CAC. Therefore, we explored whether IL-20 is involved in the infiltration of macrophages into adipose tissue. Immunofluorescence staining revealed that IL-20 colocalized with F4/80^+^ macrophages in adipose tissue following tumor inoculation (Fig. 7e). 7E treatment significantly reduced the F4/80^+^IL-20^+^ macrophage frequency in the epididymal fat tissue (Fig. 7e). We examined whether IL-20 directly induces lipolysis in adipocytes in vitro. Epididymal fat adipocytes from C57BL/6 mice were isolated and cultured in the presence of IL-20 for 1 week. The expression of the lipolytic genes ATGL and HSL, however, was not affected by IL-20 in the adipocyte cultures (Fig. 7f, g), suggesting that IL-20 may indirectly promote lipolysis via the induction of tumor-derived lipolytic factors, such as TNF-α^47^. To test this hypothesis, we treated LLC cells with mIL-20 for 24 h and then cultured the cells in fresh medium without IL-20 for another 24 h. The primary adipocytes were then incubated in conditioned medium in the presence of 7E or an anti-TNF-α antibody for 24 h. The conditioned medium from IL-20-treated LLCs increased the expression of ATGL and HSL in the adipocytes (Fig. 7h, i). Notably, the anti-IL-20 antibody was more effective than the anti-TNF-α antibody in inhibiting the paracrine effect of IL-20 driving lipolysis (Fig. 7h, i). The results suggest that IL-20 may be involved in lipolysis through the induction of TNF-α.

### IL-20R1 deficiency protected against LLC-induced CAC

We next investigated whether IL-20R1 deficiency also protects against CAC symptoms in an LLC model. LLC cells were injected subcutaneously (s.c.) into the right flank of IL-20R1^−/−^ and WT mice. Twenty-six days later, the IL-20R1^−/−^ mice showed less body weight loss than the WT mice (Fig. 7j). There was no significant difference in tumor weight between the groups (Fig. 7k). However, the IL-20R1^−/−^ mice had a greater epididymal fat mass and lower triglyceride levels than the WT mice (Fig. 7l, m). In addition, the IL-20R1^−/−^ mice were protected against adipose atrophy and immune cell infiltration into the epididymal fat pad in the LLC-induced CAC model (Fig. 7n). These results further confirm the potential therapeutic effect of IL-20 signaling blockade on CAC.

## Discussion

In this study, we demonstrated that relatively high IL-20 expression was correlated with poor survival in patients with PDAC, especially those with late-stage PDAC. IL-20 expression was also significantly correlated with PD-L1 expression in patients with PDAC. Thus IL-20 could be used as a biomarker in the clinic.

To investigate whether IL-20 directly targets tumor cells, IHC staining was performed and confirmed that IL-20 and its receptors were expressed in the tumor glands of both human and mouse PDAC specimens. In vivo, IL-20 expression was correlated with increased tumor cell proliferation and tissue fibrosis. In addition, relatively high IL-20 levels were found in the serum of both KPC mice and orthotopic model mice, indicating that IL-20 is not only involved locally in neoplastic tissue but also elevated systemically in the circulation, which may become involved in CAC.

The anti-IL-20 antibody significantly prolonged the survival of tumor-bearing mice. Human and mouse pancreatic cancer cell lines (PANC-1, BxPC-3, and PANC-02) expressed endogenous IL-20 and its receptors, and 7E inhibited the proliferation of these cell lines in vitro. In addition, IL-20R1 knockdown in KPC cells reduced tumor growth in the orthotopic model. These data indicated that IL-20 secreted by pancreatic cancer cells could shape the tumor microenvironment to favor tumor progression in an autocrine manner.

In addition to targeting tumor cell growth, M2-like macrophages were recently suggested to comprise approximately 85% of TAMs in PDAC and promote tumor immune escape by enhancing tumor fibrosis and excluding cytotoxic T cells^49,50^. In vitro, IL-20 promoted M2 polarization, which was attenuated by 7E. Blocking IL-20 with 7E also decreased the number of CD206^+^ TAMs in orthotopic tumors compared with control PBS or mIgG treatment. Furthermore, 7E could reduce fibrosis and inhibit fibrogenesis-related gene expression (αSMA, Collagen 1, Fibronectin, Vimentin, and TGF-β)^51–54^ in the orthotopic PDAC model, which implies that the paracrine function of IL-20 allows this molecule to contribute to PDAC progression as a profibrotic factor. According to our previous study, IL-20 effectively activates stellate cells, which promote tissue remodeling in liver fibrosis^39^. This finding suggests that IL-20 is a crucial profibrogenic factor and promotes the M2 polarization of TAMs to shape the tumor microenvironment toward conditions favorable to the tumor. IL-20 is also a potent angiogenic factor and promotes tumor metastasis^51–54^. Targeting IL-20 could effectively inhibit tumor growth, reduce the M2 polarization of TAMs, and alleviate fibrosis, which are all considered to contribute to unfavorable conditions for PDAC progression.

PD-L1 expression in PDAC is associated with tumor progression since PD-L1 promotes immune suppression. In PDAC tumors from patients and KPC mice, we observed that IL-20 expression significantly correlated with PD-L1 expression. In addition, IL-20 upregulated PD-L1 expression via IFN-α, which is an upstream regulator of PD-L1 produced by some types of cancer cells^40,55^. Blocking the activity of IFN-α with an antibody attenuated IL-20-induced PD-L1 expression in KPC tumor cells in vitro, indicating that IL-20 mediated PD-L1 upregulation via IFN-α. In our animal models, neutralizing IL-20 activity also decreased the expression of PD-L1 in tumors from KPC mice and orthotopic PDAC model mice.

Of note, 7E treatment also increased the numbers of CD8^+^ and PD-1^+^ T cells in tumor tissue samples from both animal models, which likely contributed to the antitumor function of 7E in restraining tumor masses. Although the results from the orthotopic PDAC model established with ASID mice revealed that 7E could inhibit tumor growth independently from T cells, we did not exclude the possibility that IL-20 blockade disrupts the immunosuppressive microenvironment of PDAC tumors by increasing cytotoxic CD8^+^ T cell accumulation. Indeed, the combination of IL-20 blockade with anti-PD-1 therapy showed remarkable antitumor efficacy, as significantly reduced tumor weights were observed for mice treated with the combination of 7E and the anti-PD-1 antibody compared to mice treated with either 7E or the anti-PD-1 antibody alone, demonstrating synergistic efficacy in PDAC. Immune checkpoint therapy targeting PD-L1 has been shown to be ineffective, as the response rate was <3.1% and there was no response to monotherapy for PDAC^10^. Therefore, combined therapies with an anti-PD-1 antibody and other antibodies or chemotherapeutic agents have been investigated and shown to be more effective than monotherapies^56–58^. Our study provided evidence that our anti-IL-20 antibody could be a potential candidate for combination therapy with an anti-PD-1 antibody in PDAC.

A recent study demonstrated that the antibody-mediated neutralization of IL-1β significantly enhanced the antitumor activity of an anti-PD-1 antibody, which was accompanied by increased tumor infiltration by CD8^+^ T cells, indicating that targeting IL-1β has a beneficial effect on PDAC therapy outcomes^59^. We previously reported that IL-1β expression was upregulated by IL-20 in glioblastoma cells and oral cancer cells^30,35^. Whether targeting IL-20 also inhibits IL-1β-mediated pancreatic oncogenesis awaits further investigation.

In the orthotopic KPC tumor model, 7E treatment or IL-20R1 knockdown not only reduced tumor growth but also alleviated cachexic symptoms, including producing reductions in the loss of body weight and fat mass. The anticachexia effect of 7E was further confirmed in an LLC-induced cachexia model, implying that 7E has therapeutic potential for treating PDAC and alleviating cachexic symptoms.

CAC reflects the activation of the host metabolism with loss of muscle mass and adipose tissue. Only a few murine tumor models have been reported to induce a cachexia-like syndrome, such as PDAC, LLC, melanoma, and prostate tumor models^60–62^. In a prostate cancer-induced cachexia study, an IL-6 antagonist alleviated weight loss but did not inhibit tumor growth^61^. In addition, in a melanoma model, a significant reduction in body weight loss was observed with IL-6 antagonist treatment^61^. By contrast, 7E not only mitigated weight loss but also attenuated tumor growth in the orthotopic PDAC model. Although IL-20 blockade did not significantly alleviate muscle wasting, it preserved adipose tissue in the animal models of CAC studied. Therefore, an IL-20 antagonist may be a relatively effective drug for treating PDAC and CAC.

In patients with CAC, the increased catabolism of lipid stores causes exhaustive loss of WAT. Inhibition of lipolysis has been shown to prevent CAC in a mouse model^19^. In the current study, mice inoculated with KPC or LLC tumor cells exhibited symptoms of cachexia, including muscle wasting and excessive loss of body weight and epididymal fat. By contrast, treatment of KPC or LLC tumor-bearing mice with IL-20 blockade prevented CAC-associated WAT lipolysis and maintained the adipose fat mass and body weight. Intriguingly, cancer-induced muscle wasting was not prevented by 7E treatment in either model, suggesting that IL-20 contributed to CAC mainly by promoting lipolysis in adipose tissues.

Our results demonstrate that IL-20 blockade by 7E is a promising treatment for PDAC with several advantages: (1) IL-20 is a prosurvival factor, and targeting IL-20 with 7E alone attenuated pancreatic cancer cell growth. (2) IL-20 blockade also shaped the microenvironment toward conditions unfavorable for tumors in multiple aspects, including the inhibition of protumorigenic M2 macrophage infiltration and alleviation of fibrosis. Aggressive fibrosis causes a hardened pancreas inaccessible to drugs. Thus the alleviation of fibrosis by IL-20 blockade might overcome this issue to improve the penetration of cytotoxic T cells or other chemotherapeutic drugs to allow killing of tumor cells. (3) IL-20 induced PD-L1 expression on PDAC tumor cells, and the combination of IL-20 blockade with anti-PD-1 therapy exhibited synergistic tumor-suppressive efficacy. (4) Targeting IL-20 not only limited tumor growth but also alleviated CAC symptoms, which profoundly improved the overall survival of mice with orthotopic PDAC tumors. Collectively, our results showed that IL-20 blockade is a potential treatment for PDAC according to the multiple protumorigenic roles of IL-20, including tumor cell proliferation, fibrosis, metastasis^28^, angiogenesis^63^, and M2 macrophage polarization. Blockade of IL-20 could lead to potentially more efficacious outcomes than targeting a single immune checkpoint (PD-L1) and overcome the limitations of currently available immunotherapies for the treatment of PDAC.

In conclusion, we demonstrated that IL-20 played pivotal roles in PDAC and that the anti-IL-20 antibody 7E retarded tumor progression, inhibited fibrosis in the pancreas, downregulated PD-L1 expression, mitigated CAC, and synergized with anti-PD-1 therapy to inhibit tumor growth. Therefore, the anti-IL-20 antibody is a potential therapeutic for PDAC.

## Methods

### Human pancreatic tissue samples

Tumor samples were taken from 72 patients with primary PDAC between January 1991 and January 2017 (stage I, *n* = 3; stage II, *n* = 38; stage III, *n* = 16; stage IV, *n* = 15). The clinicopathological variables evaluated are listed in Supplementary Table 1. Biopsies were taken after obtaining informed consent from the participants. This retrospective study was approved by the National Cheng Kung University Hospital Institutional Review Board (IRB No: A-ER-106-027). The following clinical practice guidelines for pancreatic cancer were applied: In principle, resectable diseases are treated by surgery (Whipple operation or pylorus preserving pancreatoduodectomy) with or without adjuvant chemotherapy and radiotherapy. Unresectable tumors (locally advanced or metastatic disease) are treated with chemotherapy or supportive care.

### Histological analysis and IHC staining

Hematoxylin and eosin (H&E) staining and IHC staining were performed following the standard protocols. The primary antibodies used were anti-IL-20 (7E; diluted to 5 µg/ml), anti-IL-20R1 (Catalog number: ab203196; Abcam; 1:200 dilution), anti-IL-20R2 (Catalog: 14-1206, clone: 20RNTC; eBioscience™, Thermo Fisher Scientific; diluted to 5 µg/ml), anti-IL-22R1 (Catalog: MAB42941; Clone: # 496514; R&D Systems, Minneapolis, MN; diluted to 5 µg/ml), anti-Ki-67 (Catalog: ab16667; Cone: SP6; Abcam; 1:200 dilution), anti-F4/80 (Catalog: ab6640; Abcam; 1:200 dilution), anti-CD-206 (Catalog: ab64693; Abcam; diluted to 1 µg/ml), anti-PD-L1 (Catalog: 17952-1-AP; Proteintech; 1:500 dilution), anti-CD8 (Catalog: ab217344; Abcam; 1:2000 dilution), anti-PD-1 (Catalog: ab214421; Abcam; 1:1000 dilution), anti-β-dystroglycan (Catalog: 11017-1-AP; Proteintech; 1:200 dilution), anti-ATGL (Catalog: #2138; cell signaling; 1:500 dilution), and anti-LIPE (Catalog: #OAAF00742; Aviva Systems Biology; 1:100 dilution). The secondary antibodies used were anti-human IgG (Catalog: 109-035-003; Jackson ImmunoResearch; 1:500 dilution), anti-rabbit IgG (Catalog: 111-035-003; Jackson ImmunoResearch; 1:500 dilution), and anti-rat IgG (Catalog: 405405; clone: Poly4054; BioLegend; 1:500 dilution). The anti-IL-20 mAb 7E was generated using the standard protocols^64^. 7E was confirmed to specifically recognize only IL-20, not any other members of the IL-10 family. This antibody was also validated to recognize both human and mouse IL-20 (ref. ^64^). For quantification of Ki-67^+^ proliferating cells in the pancreases of KPC mice, H&E staining and image analysis software (ImmunoRatio; https://www.medfloss.org/node/410) were used. Counterstaining was carried out with hematoxylin, and images were acquired using a digital microscope camera (DP12; Olympus Co., Tokyo, Japan). IHC images were acquired to measure the percentage of positively stained area for IL-20, PD-L1, αSMA, F4/80, CD206, CD8, and PD-1 in clinical samples and animal experiments. The percentage of positively stained area was calculated with the Tissue-Faxs software.

### Immunofluorescence staining

Immunofluorescence staining was performed according to the protocols used for IHC analysis. The primary antibodies used were anti-IL-20 (7E) and anti-PD-L1 (Catalog: 17952-1-AP; Proteintech; 1:200 dilution). The secondary antibodies used were AlexaFluor 488-conjugated anti-human (Catalog: 109-545-003; Jackson ImmunoResearch; 1:200 dilution) and AlexaFluor 594-conjugated anti-rabbit (Catalog: 111-585-003; Jackson ImmunoResearch; 1:200 dilution) antibodies.

### Isolation of pancreatic tumor cells from KPC mice

Primary pancreatic tumor cells were isolated from KPC mouse tumors. For bioluminescence imaging experiments, a luciferase-expressing derivative of primary KPC tumor cells (KPC-luc) was prepared by stably transfecting cells with pCAG-luc, which has the firefly luciferase gene encoded under the control of the (r)-actin promoter and the CMVIE enhancer, and a stable cell line was established.

### Mice and treatment

The KPC mice were maintained in C57BL/6J background with Kras^G12D^ mutation and Trp53 ablation. KPC/Luc cells (2 × 10^6^) were injected orthotopically into the pancreas of C57BL/6J and NOD.Cg-Prkdc^scid^Il2rg^tm1Wjl^/YckNarl (ASID) mice, which were obtained from the National Laboratory Animal Center (NLAC; Taiwan). Bioluminescence and fluorescence images were acquired (IVIS 50; Xenogen, Caliper Life Sciences, Hopkinton, MA) to detect luminescence from the KPC/Luc cells. Using the same approaches as those described for the s.c. model, treatments were started on day 4 and ended 4 weeks after tumor inoculation.

IL-20R1-deficient (IL-20R1^−/−^) mice were maintained on the C57BL/6J background^65^. To evaluate the effects of 7E, KPC mice (*n* = 16 per group) and orthotopic model mice (*n* = 5 per group) were randomly assigned to the indicated groups and treated with PBS, 7E (6 mg/kg, intraperitoneal (i.p.)), mIgG (6 mg/kg, i.p.), an anti-PD-1 mAb (200 μg/per mouse, i.p.; Catalog number: BE0146, BioXCell), or 7E (6 mg/kg, i.p.) plus the anti-PD-1 mAb (200 μg/per mouse) twice per week for the duration of the experiment.

LLC cells (2 × 10^6^ per mouse) were s.c. injected into the right flank of C57BL/6J WT mice. Six days after inoculation, the tumor-bearing mice were treated with PBS, 7E (6 mg/kg, i.p.), or mIgG isotype control (6 mg/kg, i.p.) twice per week for the duration of the experiment. Four weeks after inoculation, the tumors were harvested and weighed.

All animal experiments and animal care were performed according to institutional guidelines at the Laboratory Animal Center of National Cheng Kung University (NCKU) and approved by the Affidavit of Approval of Animal Use Protocol of National Cheng Kung University (IACUC Approval No: 108118, 109071).

### Enzyme-linked immunosorbent assay

IL-20 concentrations in mouse serum from tumor-bearing and healthy mice were measured using a mouse IL-20 ELISA Kit (Cat. Number: DY1204; R&D Systems) according to the manufacturer’s instructions.

### Cell proliferation assay

BxPC-3 and PANC-1 cells (4 × 10^3^) were treated with human (h) IL-20 (200 ng/ml) for 72 h in medium containing 0.5% fetal bovine serum. To confirm the specific activity of IL-20, 7E (2 μg/ml) was added to cultures, either alone or with IL-20 at a 10:1 (7E:IL-20) ratio. The cells were then incubated with 1 mg/ml MTT (Sigma-Aldrich) for 2 h, and the MTT–formazan crystals formed were dissolved in dimethyl sulfoxide (Sigma-Aldrich). Absorbance was measured at 550 nm.

### Generation of a stable IL-20R1-knockdown KPC cell line

KPC cells were infected with a lentivirus containing a pLKO1 plasmid that carried an IL-20R1-specific short hairpin RNA (shIL-20R1) sequence or nonsilencing shRNA construct (Ctrl) from RNA Technology Platform and Gene Manipulation Core (Academia Sinica, Taiwan) according to the manufacturer’s instructions. Forty-eight hours after the addition of the virus, stably transduced cells were selected by adding 2 mg/ml puromycin to the growth medium for 1 week, and then the surviving cells were collected for further applications.

### Culture of macrophages

BMDMs were isolated from the tibias of WT C57BL/6 mice^66^ and treated with monocyte colony-stimulating factor (100 ng/ml) (PeproTech, Catalog number: 315-02) for 5 days to differentiate them into macrophage-lineage cells (M0-type macrophages). Macrophages (1 × 10^6^) were treated with IL-20 (500–1000 ng/ml) for 48 h. To verify the neutralization of IL-20, 7E was added to the culture system, either alone or together with IL-20 at a 10:1 concentration ratio (7E:IL-20).

### Flow cytometry

Single-cell suspensions were prepared in ice-cold PBS containing 1% bovine serum albumin (Catalog: UR-BSA004; Sigma) and 1% sodium azide (Catalog: S2002; Sigma). Cells (1 × 10^6^) were labeled with fluorescein isothiocyanate–anti mouse F4/80 (macrophage marker; Catalog: 123107; clone: BM8; BioLegend; diluted to 2 µg/ml), allophycocyanin–anti CD-86 (M1-type macrophage marker; Catalog: 105011; clone: GL1; BioLegend; diluted to 2 µg/ml), and phycoerythrin–anti CD-206 (M2-type macrophage marker; Catalog: 141705; clone: C068C2; BioLegend; diluted to 5 µg/ml) at 4 °C for 60 min. The cells were washed three times and analyzed using a flow cytometry (FACS Canto II; BD Biosciences). FlowJo_V10 software was used for data acquisition and analysis.

### PD-L1 expression on KPC cells

To examine the expression of PD-L1 and IFN-α, KPC cells were incubated with mIL-20 (400 ng/ml) in serum-free Dulbecco’s modified Eagle’s medium for 0, 8, 16, and 24 h. 7E was used to inhibit the activity of mIL-20. KPC cells were treated with mIL-20 (400 ng/ml), mIL-20 (400 ng/ml) plus 7E (4 μg/ml), mIL-20 (400 ng/ml) plus mIgG (4 μg/ml), or mIL-20 (400 ng/ml) plus an anti-IFN-α antibody (4 μg/ml) for 24 h, and then total mRNA was isolated and analyzed.

### Skeletal muscle and adipose tissue histology

For histopathological analysis, skeletal muscle and adipose tissue samples were fixed in 4% paraformaldehyde, and the cross-sectional area (CSA) of the soleus muscle or the sizes of adipocytes in epididymal fat were analyzed. To quantify the CSA of the soleus muscle or the sizes of adipocytes in epididymal fat, five different fields were randomly chosen and analyzed using ImageJ (https://imagej.nih.gov/ij/).

### Isolation of epididymal fat adipocytes and coculture with conditioned medium

The epididymal fat pads of mice were isolated, and adipocytes were cultured following a standard protocol. To test the effect of IL-20 on lipolytic gene expression, the adipocytes were cultured in differentiation medium (1 μg/ml insulin, 0.25 μM dexamethasone, 0.5 mM 3-isobutyl-1-methylxanthine, 2.5 μM rosiglitazone, and 1 nM triiodothyronine) for 7 days and then incubated with mIL-20 (200 ng/ml) or 7E (2 μg/ml) for 24 h.

To evaluate the effect of IL-20 on tumor-derived lipolytic factors, LLC cells were pretreated with mIL-20 (200 ng/ml) for 24 h. Then the medium was replaced with fresh medium without mIL-20. The conditioned medium was collected 24 h later and added to adipocyte cultures with 7E (2 μg/ml), mIgG (2 μg/ml; Invitrogen), rat IgG (2 μg/ml; BioLegend), or an anti-TNF-α antibody (2 μg/ml; BioLegend).

### Real-time quantitative polymerase chain reaction

Total RNA was isolated. Reverse transcription was performed with a reverse transcriptase (Thermo Fisher). cDNA was then amplified on a thermocycler (LightCycler 480; Roche Diagnostics, Indianapolis, IN) with SYBR Green I (Roche Diagnostics). The quantitative results for mRNA were normalized to the results for glyceraldehyde 3-phosphate dehydrogenase, the control housekeeping gene. Relative changes in mRNA expression were determined by the 2^−ΔΔCt^ method. The primer sequences are listed in Supplementary Table 2.

### Statistical analysis

Statistical analysis was performed with GraphPad Prism version 6 (GraphPad Software, San Diego, CA, USA). Experimental data are presented as the mean ± SD/SEM. GraphPad Prism software was employed to process initial data and plot graphs. The endpoints analyzed were the survival of patients with low or high IL-20 expression and that of KPC mice treated with PBS, mIgG, or 7E, which were evaluated using Kaplan–Meier methods. The Cox proportional hazards model was used for univariate analyses of disease prognosis. Pearson correlation analysis was used for evaluation of the relationship between IL-20 and PD-L1 in clinical specimens. Two-group comparisons were analyzed by a two-sided Student’s *t* test. One-way analysis of variance was used to compare data among groups in experiments including three or more groups. *p* < 0.05 was considered significant (*p < 0.05, **p < 0.01, ***p < 0.001, and ****p < 0.0001).

### Reporting summary

Further information on research design is available in the Nature Research Reporting Summary linked to this article.

## Supplementary information

Supplementary information

Reporting Summary

Data Source

## Data Availability

The source data underlying Figs. 1–7 and Supplementary Figs. 1–5 are provided with the paper as a Source data file. All remaining relevant data are available in the article, supplementary information, or from the corresponding author upon reasonable request.
