# Supplementary material for: IL-20 antagonist suppresses PD-L1 expression and prolongs survival in pancreatic cancer models
Source: Nat Commun. 2020 Sep 14;11:4611. doi: 10.1038/s41467-020-18244-8 (PMC7490368; doi:10.1038/s41467-020-18244-8)
Supplement: Supplementary file 1 — Supplementary information [file 41467_2020_18244_MOESM1_ESM.pdf]

Supplementary information

IL-20 antagonist suppresses PD-L1 expression and prolongs survival in pancreatic cancer models

Lu et al.

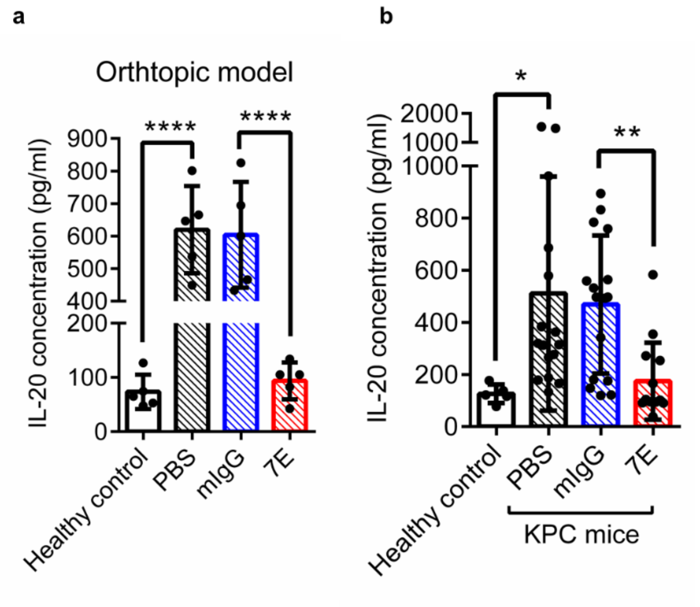

Supplementary Figure 1. IL-20 was upregulated in the serum of PDAC mouse model.

Serum levels of IL-20 in (a) orthotopic model (n=5) and (b) KPC mice (n=16) treated with PBS, mIgG, or 7E. Statistical significance was determined by one-way ANOVA ((a), p=0.0394; (b), p<0.0001), followed by Sidak's multiple comparisons test between groups test. Values are means ± SD. *p<0.05, **p<0.01, ****p < 0.0001. The experiments in (a) and (b) were repeated three times independently with similar results, and the data of one representative experiment are shown.

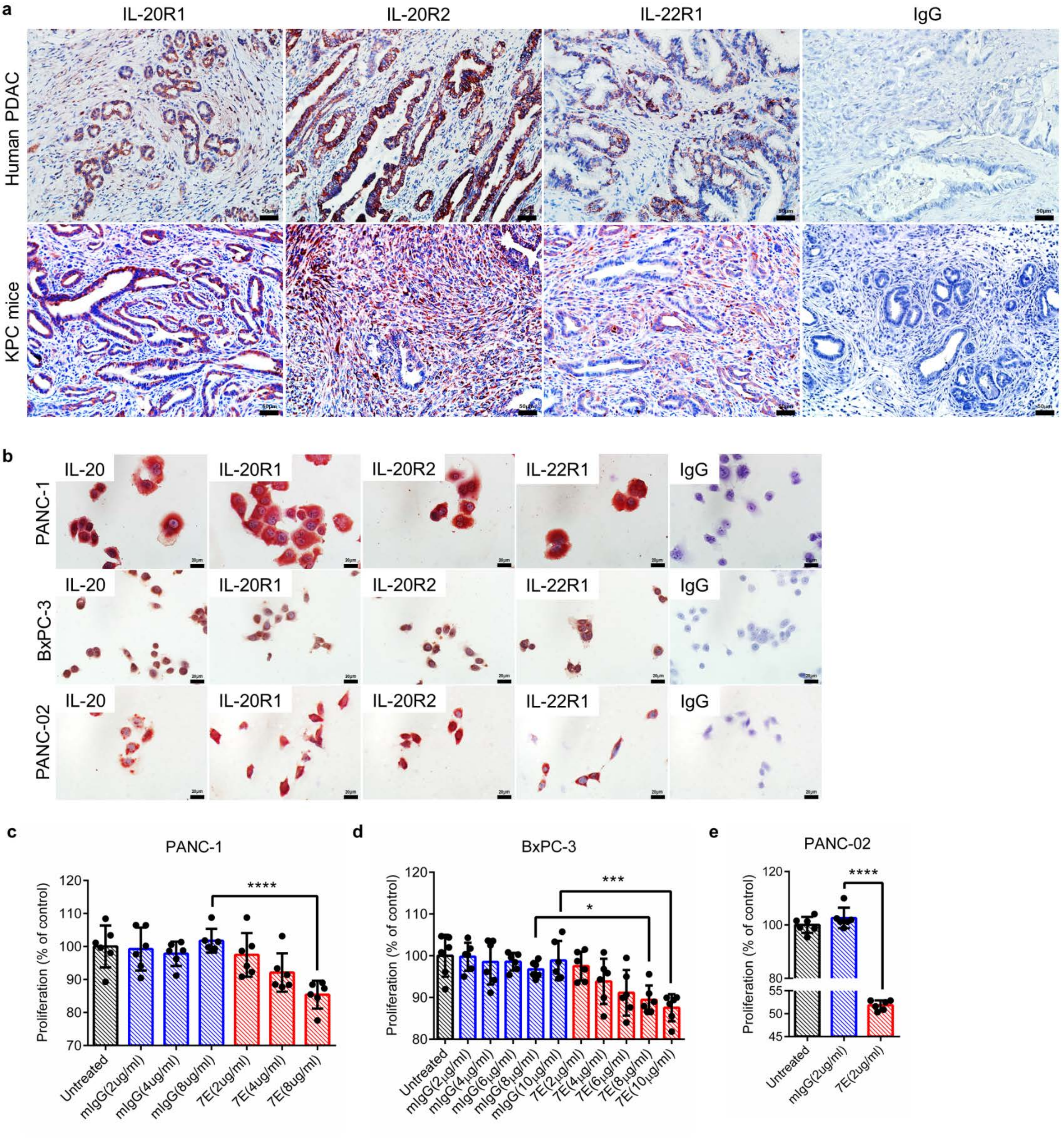
a
IL-20R1
IL-20R2
IL-22R1
IgG
Human PDAC
KPC mice
b
PANC-1
BxPC-3
PANC-02
IL-20
IL-20R1
IL-20R2
IL-22R1
IgG
c
PANC-1
****
Proliferation (% of control)
Untreated
mIgG(2ug/ml)
mIgG(4ug/ml)
mIgG(8ug/ml)
7E(2ug/ml)
7E(4ug/ml)
7E(8ug/ml)
d
BxPC-3
***
*
Proliferation (% of control)
Untreated
mIgG(2μg/ml)
mIgG(4μg/ml)
mIgG(6μg/ml)
mIgG(8μg/ml)
mIgG(10μg/ml)
7E(2μg/ml)
7E(4μg/ml)
7E(6μg/ml)
7E(8μg/ml)
7E(10μg/ml)
e
PANC-02
****
Proliferation (% of control)
Untreated
mIgG(2ug/ml)
7E(2ug/ml)

Supplementary Figure 2. 7E inhibited cell proliferation in PANC-1 and BxPC-3 cells.

(**a**) Pancreatic cancer specimens of human and mouse were stained with anti–IL-20R1, –IL-20R2, and –IL-22R1 mAb. Original magnification 200×; Scale bar, 50 μm. (**b**) Staining of IL-20 and its receptors (IL-20R1, IL-20R2, and IL-22R1) in human PANC-1 and BxPC-3 cells and mouse PANC-02 cells. Original magnification 400×; Scale bar, 20 μm. (**c-e**) The MTT assay demonstrated that cell proliferation was inhibited in 7E-treated PANC-1, BxPC-3, and PANC-02 cells. Statistical significance was determined by one-way ANOVA ((**a**), $p=0.0001$; (**b**), $p<0.0001$; (**c**), $p<0.0001$), followed by Sidak's multiple comparisons test between groups test. *$p<0.05$, ***$p < 0.001$, ****$p<0.0001$ compared with mIgG-treated controls. Data are means ± SD of six repeated samples. The experiments in (a-e) were repeated three times independently with similar results, and the data of one representative experiment are shown.

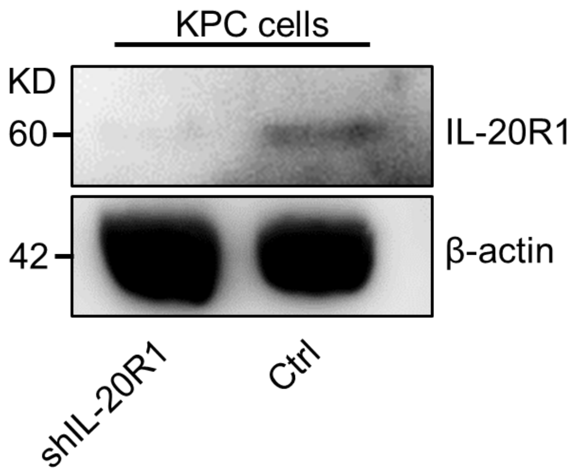

Supplementary Figure 3. Inhibition of IL-20R1 expression on KPC cells. IL-20R1 suppression in KPC cells stably transfected with IL-20R1 shRNA or control shRNA was analyzed by Western blots. The experiments in Fig. 3 was repeated three times independently with similar results, and the data of one representative experiment are shown.

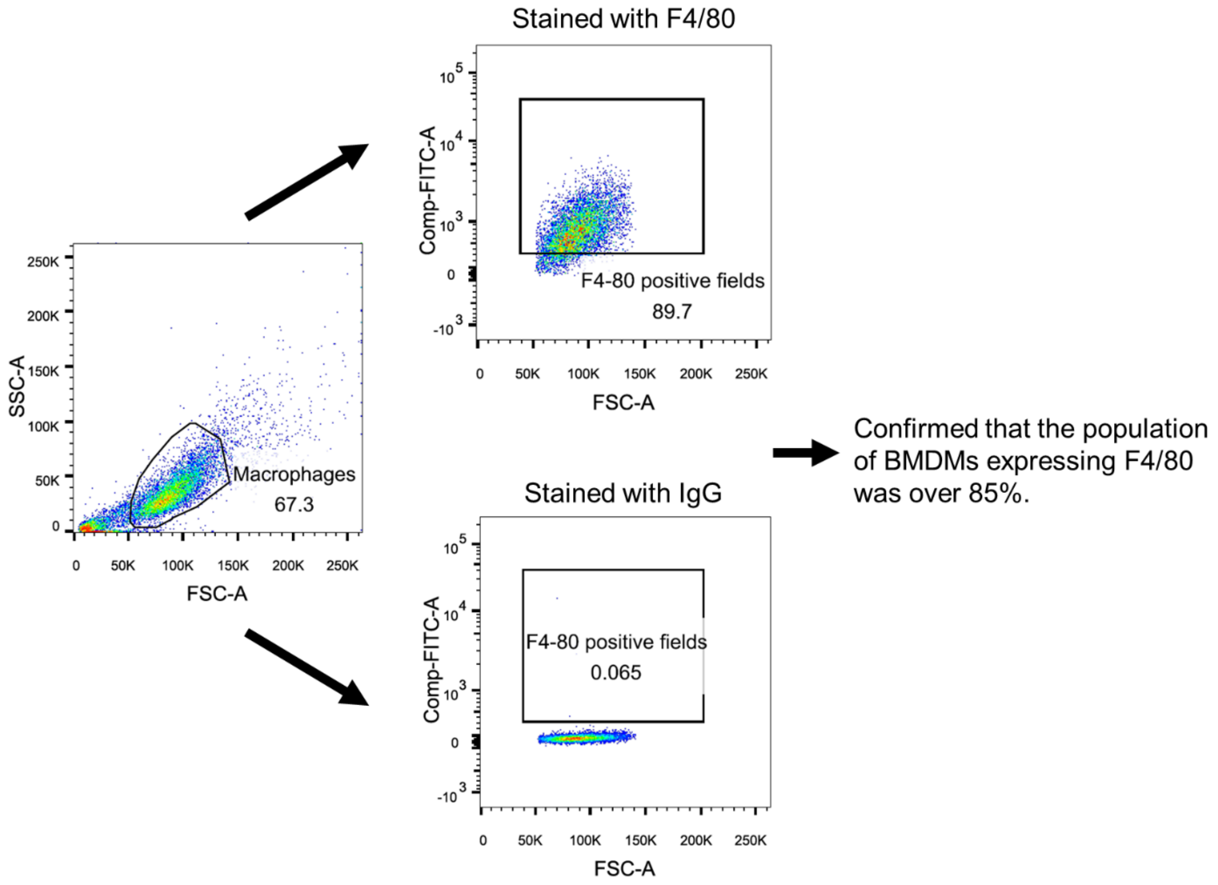

Supplementary Figure. 4 Gating strategy for quantifying the abundance of F4/80$^+$ cells in the BMDMs population. The live cells were first gated on the BMDMs population on the FSC/SSC plot. Then the BMDMs were determined based on staining for F4/80. Subsequent characteristics were analyzed within this population.

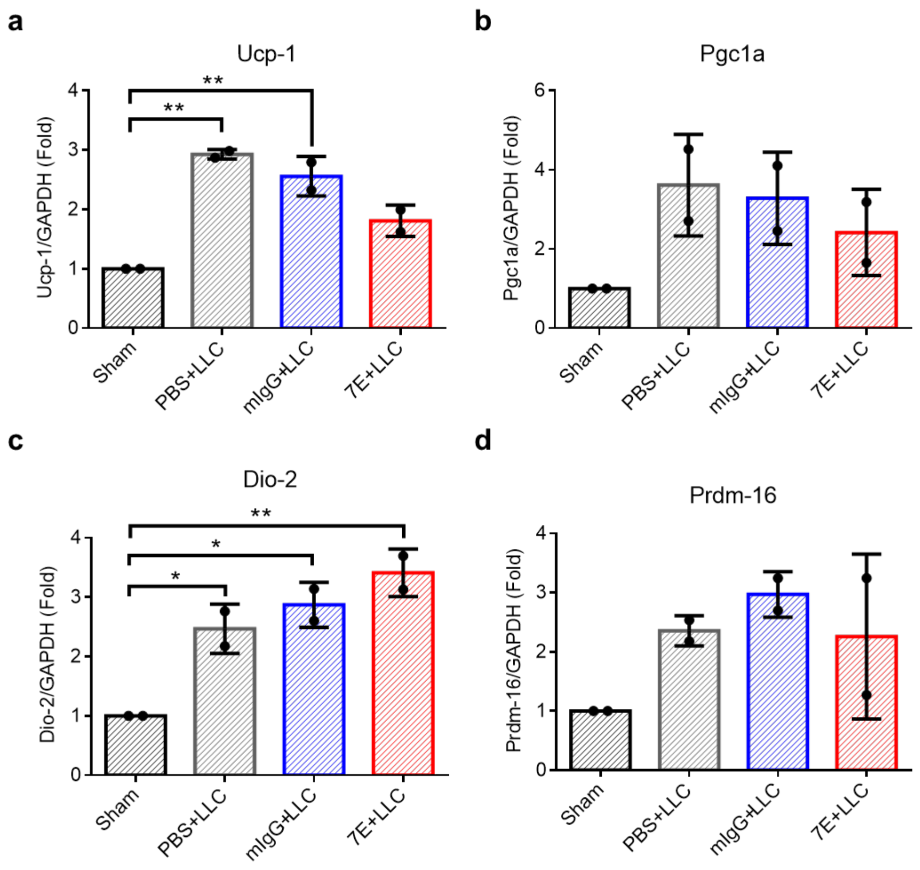

Supplementary Figure 5. The expression of WAT browning-related genes in the epididymal fat of cachectic mice.

LLC tumor-bearing mice were injected (i.p.) with PBS, mIgG (6 mg/kg), or 7E (6 mg/kg) (n = 8 in each group) twice a week throughout the study. Sham controls (n = 8) were not injected with cancer cells. The mRNA transcripts of (**a**) Ucp-1 (One-way ANOVA, p=0.0031), (**b**) Pgc-l-a, (**c**) Dio-2 (One-way ANOVA, p=0.0031), and (**d**) Prdm-16 in the epididymal fat of the cachectic mice were analyzed using RT-qPCR with specific primers. GAPDH was an input control. The experiments in (a), (b), (c), and (d) were repeated three times independently with similar results, and the data of one representative experiment are shown. Data are means ± SD. *p < 0.05, **p < 0.01 compared with sham control.

Supplementary Tables

Supplementary Table 1-Correlations between IL-20 expression and various clinicopathological parameters

Parameters		Total number	Univariate P Value
**Age**			
<60		23	0.7184
≧60		49	
**Sex**			
Male		41	0.6378
Female		31	
**Differentiation**			
Well/Moderately differentiated		63	0.2293
Poorly differentiated		9	
**Level of IL-20**			
High expression [b]		26	0.0424 [c]
Low expression [a]		46	
**PDAC Stage**			
Early stage	Stage I	3	0.0122 [c]
	Stage II	38	
Late stage	Stage III	16	
	Stage IV	15	

- [a] Low expression: staining positive area < 5%
- [b] High expression: staining positive area ≥ 5%
- [c] Statistically significant

Supplementary Table 2- A list of primers used in this study

**Gene**	**Forward (5´ to 3´)**	**Reverse (5´ to 3´)**
m-αSMA	ACTGGGACGACATGGAAAAG	GAAGGAATAGCCACGCTCAG
m-Collagen	CTGCAAGAACAGCATTGCAT	GGCGTGATGGCTTATTTGTT
m-Fibronectin	TGTGACAACTGCCGTAGACC	ATGAAGCACTCAATGGGGCA
m-Vimentin	ATGTGGACGTTTCCAAGCCT	ACCTGTCTCCGGTACTCGTT
m-TGF-β	AGCAGCAACCGACTGAAGAA	GCATGTAGAGAGCGGAGCA
m-PD-L1	TAATCAGCTACGGTGGTGCG	AAACATCATTCGCTGTGGCG
m-IFN-α	GATTCCCACAGGAGAAGGTGG	AGCTCACTCAGGACAGGGAT
m-Ucp-1	AAGCTGTGCGATGTCCATGT	AAGCCACAAACCCTTTGAAAA
m-Prdm-16	GCACGGTGAAGCCATTCATATG	TCGGCGTGCATCCGCTTGTG
m-Dio-2	GTCCGCAAATGACCCCTTT	CCCACCCACTCTCTGACTTTC
m-Pgc-1a	AGACAAATGTGCTTCGAAAAAGAA	GAAGAGATAAAGTTGTTGGTTTGGC
m-ATGL	CAGCACATTTATCCCGGTGTAC	AAATGCCGCCATCCACATAG
m-HSL	GCTGGAGGAGTGTTTTTTTGC	AGTTGAACCAAGCAGGTCACA
