# Supplementary material for: IL-20 antagonist suppresses PD-L1 expression and prolongs survival in pancreatic cancer models
Source: Nat Commun. 2020 Sep 14;11:4611. doi: 10.1038/s41467-020-18244-8 (PMC7490368; doi:10.1038/s41467-020-18244-8)
Supplement: Supplementary file 2 — Reporting Summary [file 41467_2020_18244_MOESM2_ESM.pdf]

nature research

Corresponding author(s): Ming-Shi, Chang

Last updated by author(s): Jul 17, 2020

Reporting Summary

Nature Research wishes to improve the reproducibility of the work that we publish. This form provides structure for consistency and transparency in reporting. For further information on Nature Research policies, see Authors & Referees and the Editorial Policy Checklist .

Statistics

For all statistical analyses, confirm that the following items are present in the figure legend, table legend, main text, or Methods section.

n/a	Confirmed	
☐	☒	The exact sample size (*n*) for each experimental group/condition, given as a discrete number and unit of measurement
☐	☒	A statement on whether measurements were taken from distinct samples or whether the same sample was measured repeatedly
☐	☒	The statistical test(s) used AND whether they are one- or two-sided<br>*Only common tests should be described solely by name; describe more complex techniques in the Methods section.*
☒	☐	A description of all covariates tested
☐	☒	A description of any assumptions or corrections, such as tests of normality and adjustment for multiple comparisons
☐	☒	A full description of the statistical parameters including central tendency (e.g. means) or other basic estimates (e.g. regression coefficient) AND variation (e.g. standard deviation) or associated estimates of uncertainty (e.g. confidence intervals)
☐	☒	For null hypothesis testing, the test statistic (e.g. *F*, *t*, *r*) with confidence intervals, effect sizes, degrees of freedom and *P* value noted<br>*Give P values as exact values whenever suitable.*
☒	☐	For Bayesian analysis, information on the choice of priors and Markov chain Monte Carlo settings
☒	☐	For hierarchical and complex designs, identification of the appropriate level for tests and full reporting of outcomes
☐	☒	Estimates of effect sizes (e.g. Cohen's *d*, Pearson's *r*), indicating how they were calculated

Our web collection on statistics for biologists contains articles on many of the points above.

Software and code

Policy information about availability of computer code

Data collection	BD FACSDiva software version 8.0.1 (BD Biosciences)
Data analysis	HistoQuest (version 4.0), FlowJo V10; GraphPad Prism V6 (GraphPad Software Inc.); ImmunoRatio software (version 1); ImageJ software (version 1.52)

For manuscripts utilizing custom algorithms or software that are central to the research but not yet described in published literature, software must be made available to editors/reviewers. We strongly encourage code deposition in a community repository (e.g. GitHub). See the Nature Research guidelines for submitting code & software for further information.

Data

Policy information about availability of data

All manuscripts must include a data availability statement. This statement should provide the following information, where applicable:

- Accession codes, unique identifiers, or web links for publicly available datasets
- A list of figures that have associated raw data
- A description of any restrictions on data availability

All the data supporting the findings of this study are available within the article and its supplementary information files.

Field-specific reporting

Please select the one below that is the best fit for your research. If you are not sure, read the appropriate sections before making your selection.

☒ Life sciences ☐ Behavioural & social sciences ☐ Ecological, evolutionary & environmental sciences

Life sciences study design

All studies must disclose on these points even when the disclosure is negative.

Sample size	Sample sizes for all animal experiments were determined using past experience, usually with 5 animals or more per experimental group in a given experiment. (see i.g Chiu, Yi-Shu et al. Scientific reports vol. 7,1 17609. 14 Dec. 2017; Hsu, Yu-Hsiang et al, Journal of immunology, vol. 188,4 (2012): 1981-91).
Data exclusions	No data was excluded.
Replication	All data have been successfully reproduced at least three times, as described in each figure legends.
Randomization	Samples were randomized before starting treatment in all of in vivo and in vitro experiments.
Blinding	Scoring of histological slides was blinded. For the rest of the experiments no blinding was deemed necessary as no subjective assessments were necessary.

Reporting for specific materials, systems and methods

We require information from authors about some types of materials, experimental systems and methods used in many studies. Here, indicate whether each material, system or method listed is relevant to your study. If you are not sure if a list item applies to your research, read the appropriate section before selecting a response.

Materials & experimental systems

n/a	Involved in the study	
☐	☒	Antibodies
☐	☒	Eukaryotic cell lines
☒	☐	Palaeontology
☐	☒	Animals and other organisms
☐	☒	Human research participants
☒	☐	Clinical data

Methods

n/a	Involved in the study	
☒	☐	ChIP-seq
☐	☒	Flow cytometry
☒	☐	MRI-based neuroimaging

Antibodies

Antibodies used

Antibodies used for IHC:

Anti-IL-20 (7E; diluted to 5μg/ml), anti-IL-20R1(Catalog: ab203196 ; Abcam; 1:200 dilution), anti-IL-20R2(Catalog: 14-1206, clone: 20RNTC; eBioscience™, Thermo Fisher Scientific; diluted to 5μg/ml), anti-IL-22R1 mAb (Catalog: MAB42941; Clone: # 496514; R&D Systems, Minneapolis, MN; diluted to 5μg/ml), anti-Ki-67 (Catalog: ab16667; Cone: SP6; Abcam; 1:200 dilution), anti-F4/80 (Catalog: ab6640; Abcam; 1:200 dilution), anti-CD-206 (Catalog: ab64693 ; Abcam; diluted to 1μg/ml), anti-PD-L1 (Catalog: 17952-1-AP; Proteintech; 1:500 dilution), anti-CD8 (Catalog: ab217344 ; Abcam; 1:2000 dilution), anti-PD-1 (Catalog: ab214421; Abcam; 1:1000 dilution), anti-β-dystroglycan (Catalog: 11017-1-AP; Proteintech; 1:200 dilution), anti-ATGL (Catalog: #2138; cell signaling; 1:500 dilution), anti-αSMA (Catalog: ab124964; Cone: EPR5368; Abcam; 1:1000 dilution), or anti-LIPE (Catalog: #OAAF00742; Aviva Systems Biology; 1:100 dilution). Secondary antibody: anti-human IgG (Catalog: 109-035-003; Jackson ImmunoResearch; 1:500 dilution), anti-rabbit IgG (Catalog: 111-035-003; Jackson ImmunoResearch; 1:500 dilution), anti-rat IgG (Catalog: 405405; clone: Poly4054; BioLegend; 1:500 dilution).

Antibodies used for immunofloresence:

Anti-IL-20 (7E; diluted to 5μg/ml), anti-PD-L1 (Catalog: 17952-1-AP; Proteintech; 1:200 dilution), Alexa Fluor® 488 AffiniPure Goat Anti-Human secondary antibody (Catalog: 109-545-003; Jackson ImmunoResearch; 1:200 dilution), and Alexa Fluor® 594 AffiniPure Goat Anti-Rabbit secondary antibody (Catalog: 111-585-003; Jackson ImmunoResearch; 1:200 dilution).

Antibodies used for flow cytometry:

FITC-anti mouse F4/80 (Macrophage marker; Catalog: 123107; clone: BM8; BioLegend; diluted to 2μg/ml), APC-anti CD-86 (M1-type macrophage marker; Catalog: 105011 ; clone: GL1; BioLegend; diluted to 2μg/ml), and PE- anti CD-206 (M2-type macrophage marker; Catalog: 141705; clone: C068C2 ;BioLegend; diluted to 5μg/ml).

Validation

Each antibody that was used in applications that were validated by the manufacturer per their product information

The anti-IL-20 monoclonal antibody 7E was generated using standard protocols. The specificity of 7E was confirmed to recognize only IL-20, not any other members of IL-10 family. It was also validated to recognize both human and mouse IL-20 as previously described (Clinical Immunology, Volume 117, Issue 1, 2005, Pages 65-72, ISSN 1521-6616,).

Eukaryotic cell lines

Policy information about cell lines

Cell line source(s)	Cell lines were obtained from ATCC (Human pancreatic cancer cell line: PANC-1, BxPC-3.). Mouse pancreatic cancer cell line PANC-02 was provided by Dr. Tze-Sing Huang. IL-20R1 KPC knockdown cell lines were generated as described in the manuscript.
Authentication	The cell lines used were not authenticated.
Mycoplasma contamination	The cell lines used were not tested for mycoplasma contamination.
Commonly misidentified lines (See ICLAC register)	No commonly misidentified cell lines was used in the study.

Animals and other organisms

Policy information about studies involving animals; ARRIVE guidelines recommended for reporting animal research

Laboratory animals	The LSL-KrasG12D; Trp53flox/flox; Pdx-1-Cre (KPC) mice maintained in C57BL/6J background, wild type C57BL/6J, and IL-20R1-deficient (IL-20R1–/–) mice maintained in C57BL/6J background were used in the study. Male 6-8 week old mice were used. Male ASID (ASID; NOD.Cg-PrkdcscidIl2rgtm1Wjl/YckNarl) mice between 6-8 weeks of age were purchased from National Laboratory Animal Center (NLAC), Taiwan. They were housed in cages of no more than 5 mice/cage at a temperature of 22+/- 1 degree C and relative humidity of 55+/-10% with 13 hr / 11 hr light/dark cycle.
Wild animals	No wild animals were used in this study.
Field-collected samples	There are no samples collected from the field in this study.
Ethics oversight	All animal experiments and animal care were performed according to institutional guidelines at the Laboratory Animal Center of National Cheng Kung University (NCKU), and approved by the Affidavit of Approval of Animal Use Protocol of National Cheng Kung University (IACUC Approval No: 108118, 109071).

Note that full information on the approval of the study protocol must also be provided in the manuscript.

Human research participants

Policy information about studies involving human research participants

Population characteristics	The clinicopathological parameters of patient are described in Supplementary Table 1. No covariates were used in this study.
Recruitment	We applied for 72 cases of human pancreatic cancer specimens diagnosed as pancreatic cancer from 1991 to 2017 from the Department of Pathology, National Cheng Kung University Hospital.
Ethics oversight	This retrospective study was approved by the National Cheng Kung University Hospital Institutional Review Board (IRB No: A-ER-106-027).

Note that full information on the approval of the study protocol must also be provided in the manuscript.

Flow Cytometry

Plots

Confirm that:

- [x] The axis labels state the marker and fluorochrome used (e.g. CD4-FITC).
- [x] The axis scales are clearly visible. Include numbers along axes only for bottom left plot of group (a 'group' is an analysis of identical markers).
- [x] All plots are contour plots with outliers or pseudocolor plots.
- [x] A numerical value for number of cells or percentage (with statistics) is provided.

Methodology

Sample preparation	Bone Marrow-Derived Macrophages (BMDMs) preparation is described in detail in the Material and Methods.
Instrument	Samples were collected on a flow cytometer. (BD FACSCanto™ II; BD Biosciences)
Software	The data were collected by using BD FACSDiva software (BD Biosciences) and analyzed with FlowJo V10 software (LLC).
Cell population abundance	The purity was determined by evaluation of the FSC/SSC plot along with staining with the indicated antibodies.

Gating strategy	The live cells were first gated on the BMDMs population on the FSC/SSC plot. Then the BMDMs were determined based on staining for F4/80. Subsequent characteristics were analyzed within this population.

- [x] Tick this box to confirm that a figure exemplifying the gating strategy is provided in the Supplementary Information.
